# The genome sequence of the Rivulet moth, *Perizoma affinitatum *(Stephens, 1831)

**DOI:** 10.12688/wellcomeopenres.21016.1

**Published:** 2024-03-04

**Authors:** Gavin R. Broad, Laura Sivess, Chris Fletcher, Inez Januszczak, Stephanie Holt, Dominic Phillips, Natural History Museum Genome Acquisition Lab, Darwin Tree of Life Barcoding collective, Wellcome Sanger Institute Tree of Life Management, Samples and Laboratory team, Wellcome Sanger Institute Scientific Operations: Sequencing Operations, Wellcome Sanger Institute Tree of Life Core Informatics team, Tree of Life Core Informatics collective, Darwin Tree of Life Consortium

**Affiliations:** 1 Natural History Museum, London, England, UK

**Keywords:** Perizoma affinitatum, Rivulet moth, genome sequence, chromosomal, Lepidoptera

## Abstract

We present a genome assembly from an individual male *Perizoma affinitatum* (the Rivulet moth; Arthropoda; Insecta; Lepidoptera; Geometridae). The genome sequence is 357.7 megabases in span. Most of the assembly is scaffolded into 25 chromosomal pseudomolecules, including the Z sex chromosome. The mitochondrial genome has also been assembled and is 15.9 kilobases in length.

## Species taxonomy

Eukaryota; Opisthokonta; Metazoa; Eumetazoa; Bilateria; Protostomia; Ecdysozoa; Panarthropoda; Arthropoda; Mandibulata; Pancrustacea; Hexapoda; Insecta; Dicondylia; Pterygota; Neoptera; Endopterygota; Amphiesmenoptera; Lepidoptera; Glossata; Neolepidoptera; Heteroneura; Ditrysia; Obtectomera; Geometroidea; Geometridae; Larentiinae; *Perizoma*; *Perizoma affinitatum* (Stephens, 1831) (NCBI:txid934816).

## Background

The Rivulet ( *Perizoma affinitatum*) is a Geometrid moth in the Larentiinae subfamily, with a forewing length measuring between 12–15 mm. *Perizoma affinitatum* can be identified by the prominent white cross-band along the dark grey-brown forewing, broken by a thin dark line running through. *Perizoma alchemillata* has similar markings but *P. affinitatum* can be distinguished most readily by its larger size and usually a single deep U-shape within the proximal margin of the cross band, as opposed to a double indentation. The two species may also be determined by differences in male and female genitalia ( British Lepidoptera, 2023; Waring *et al.*, 2017).

*Perizoma affinitatum* is a bivoltine species, found on the wing from May to July and August-September, overwintering underground as a pupa, emerging as larva July to Early September. It is on the wing from dusk near its foodplant, coming to light in small numbers ( Waring *et al.*, 2017).

The preferred foodplant of *P. affinitatum* is red campion ( *Silene dioica*) but they have been recorded on other *Silene* species ( Waring *et al.*, 2017), the larvae can be found living inside seed capsules of food plants, feeding on the seeds.

A common resident and well distributed in southern England, southern Scotland, Wales, Anglia and the west Midlands, with patchy distribution through the rest of England. It is local in northern mainland Scotland, northern Ireland and the Inner Hebrides ( GBIF Secretariat, 2023). Usually found in open woodland, hedgerows and chalk downland, occasionally recorded on well-vegetated sea-cliffs ( Waring *et al.*, 2017). Both the distribution and occurrence of *P. affinitatum* showed a significant decrease over a 35-year period from 1968 to 2002 in Britain potentially related to its host plant specialism ( Conrad *et al.*, 2006).

Previous studies have compared the DNA barcodes of *Perizoma affinitatum* with related species with a clear separation in morphology ( *Perizoma hydrata*) and found that these species’ barcodes do not differ by much. However, they can still be differentiated by two haplotypes with a 0.15% and 0.71% difference respectively. Due to this it is possible that the species were either of recent origin or shared mitochondrial exchanges in the Pleistocene. Investigating the full genome sequences of such families can give us an insight into how early each species diverged from one another giving us insights into the origins of such taxa ( Hausmann *et al.*, 2011).

The genome of *Perizoma affinitatum* was sequenced as part of the Darwin Tree of Life Project, a collaborative effort to sequence all named eukaryotic species in the Atlantic Archipelago of Britain and Ireland. Here we present a chromosomally complete genome sequence for *Perizoma affinitatum*, based on two specimens from Gilbert White’s House, Selborne, Hampshire, UK.

## Genome sequence report

**Figure 1.**
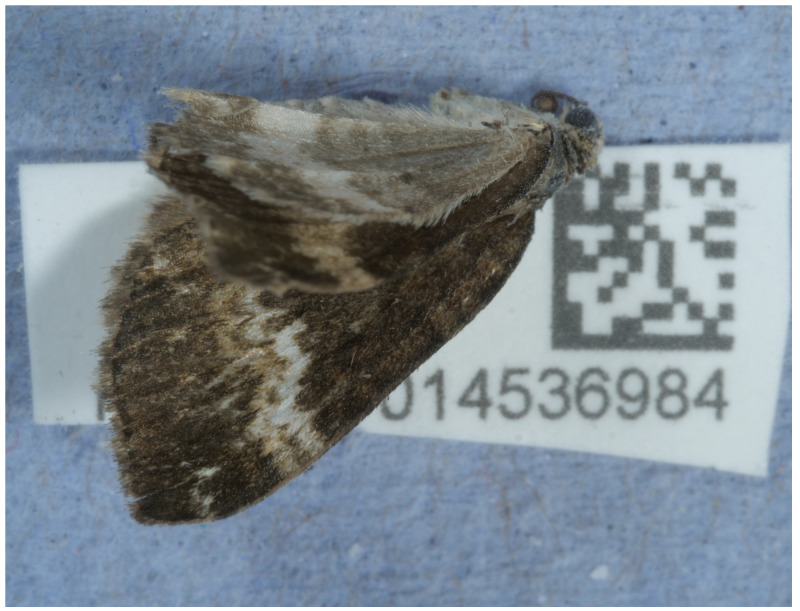
Photograph of the *Perizoma affinitatum* (ilPerAffn1) specimen used for genome sequencing.

The genome was sequenced from one male *Perizoma affinitatum* ( Figure 1) collected from Gilbert White's House, Selborne, England, UK (51.09, –0.94). A total of 68-fold coverage in Pacific Biosciences single-molecule HiFi long reads was generated. Primary assembly contigs were scaffolded with chromosome conformation Hi-C data. Manual assembly curation corrected 4 missing joins or mis-joins and removed 2 haplotypic duplications, reducing the scaffold number by 10.34%.

**Figure 2.**
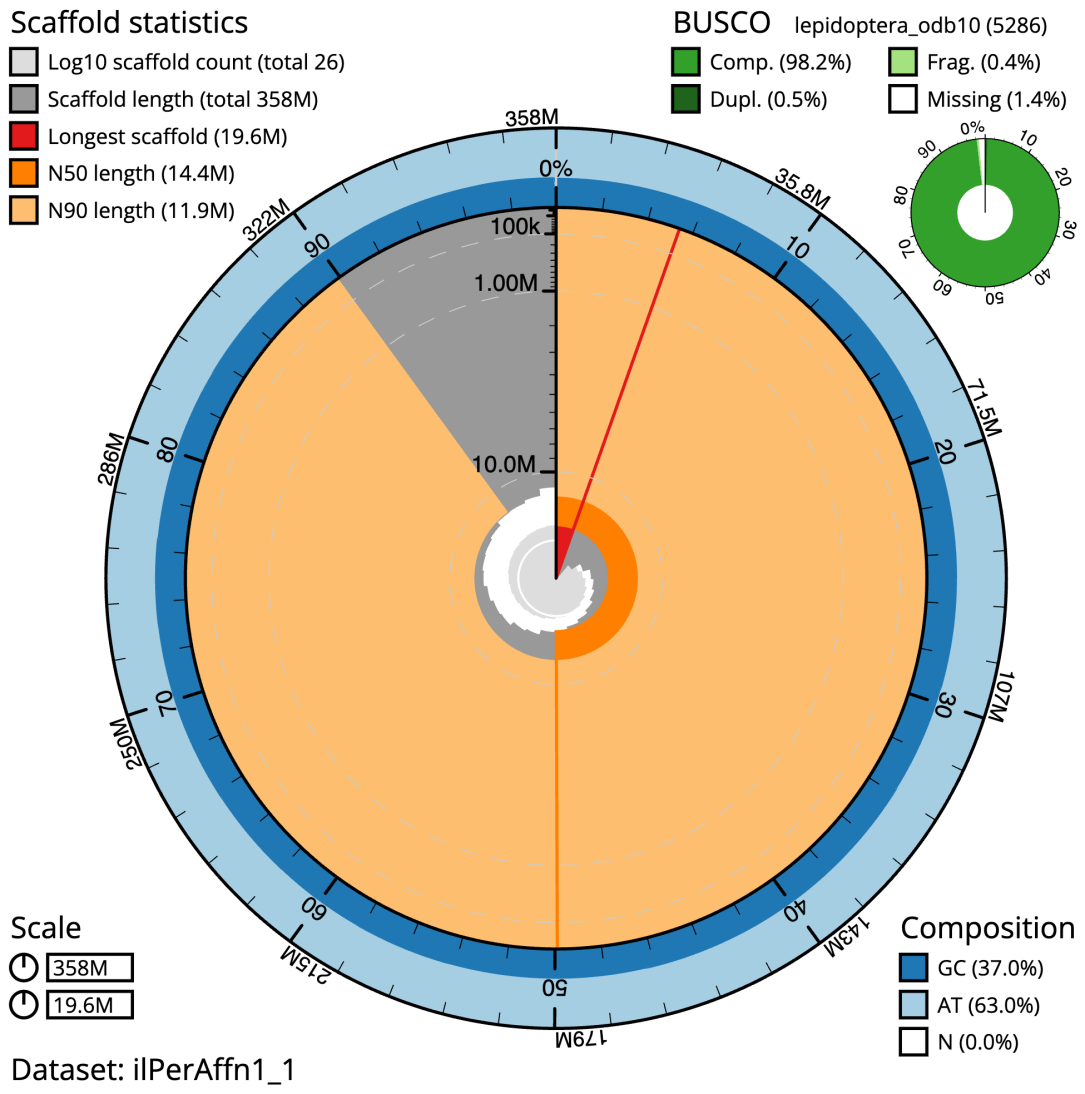
Genome assembly of *Perizoma affinitatum*, ilPerAffn1.1: metrics. The BlobToolKit Snailplot shows N50 metrics and BUSCO gene completeness. The main plot is divided into 1,000 size-ordered bins around the circumference with each bin representing 0.1% of the 357,708,599 bp assembly. The distribution of scaffold lengths is shown in dark grey with the plot radius scaled to the longest scaffold present in the assembly (19,648,534 bp, shown in red). Orange and pale-orange arcs show the N50 and N90 scaffold lengths (14,385,535 and 11,939,442 bp), respectively. The pale grey spiral shows the cumulative scaffold count on a log scale with white scale lines showing successive orders of magnitude. The blue and pale-blue area around the outside of the plot shows the distribution of GC, AT and N percentages in the same bins as the inner plot. A summary of complete, fragmented, duplicated and missing BUSCO genes in the lepidoptera_odb10 set is shown in the top right. An interactive version of this figure is available at https://blobtoolkit.genomehubs.org/view/ilPerAffn1_1/dataset/ilPerAffn1_1/snail.

**Figure 3.**
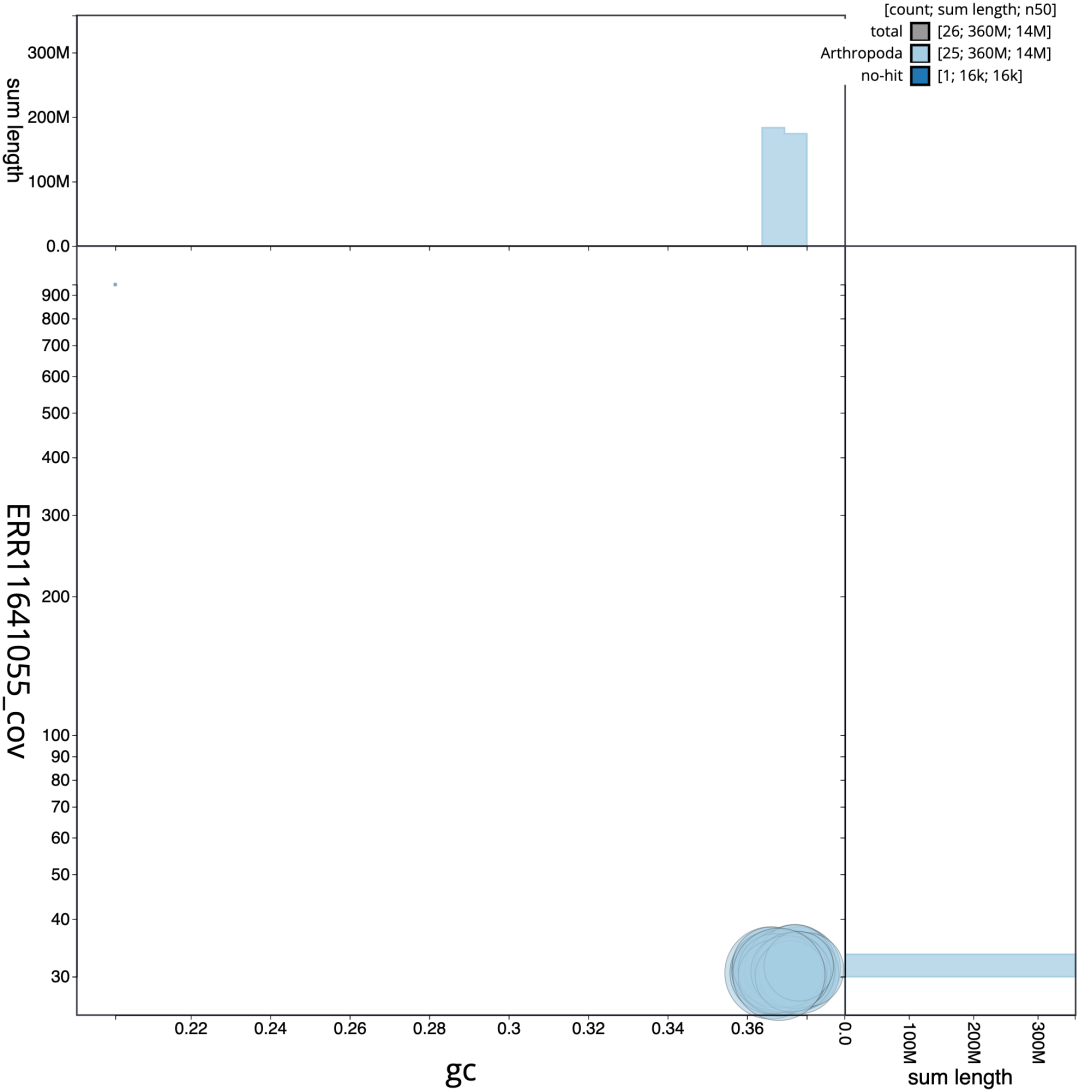
Genome assembly of *Perizoma affinitatum*, ilPerAffn1.1: BlobToolKit GC-coverage plot. Scaffolds are coloured by phylum. Circles are sized in proportion to scaffold length. Histograms show the distribution of scaffold length sum along each axis. An interactive version of this figure is available at https://blobtoolkit.genomehubs.org/view/ilPerAffn1_1/dataset/ilPerAffn1_1/blob.

**Figure 4.**
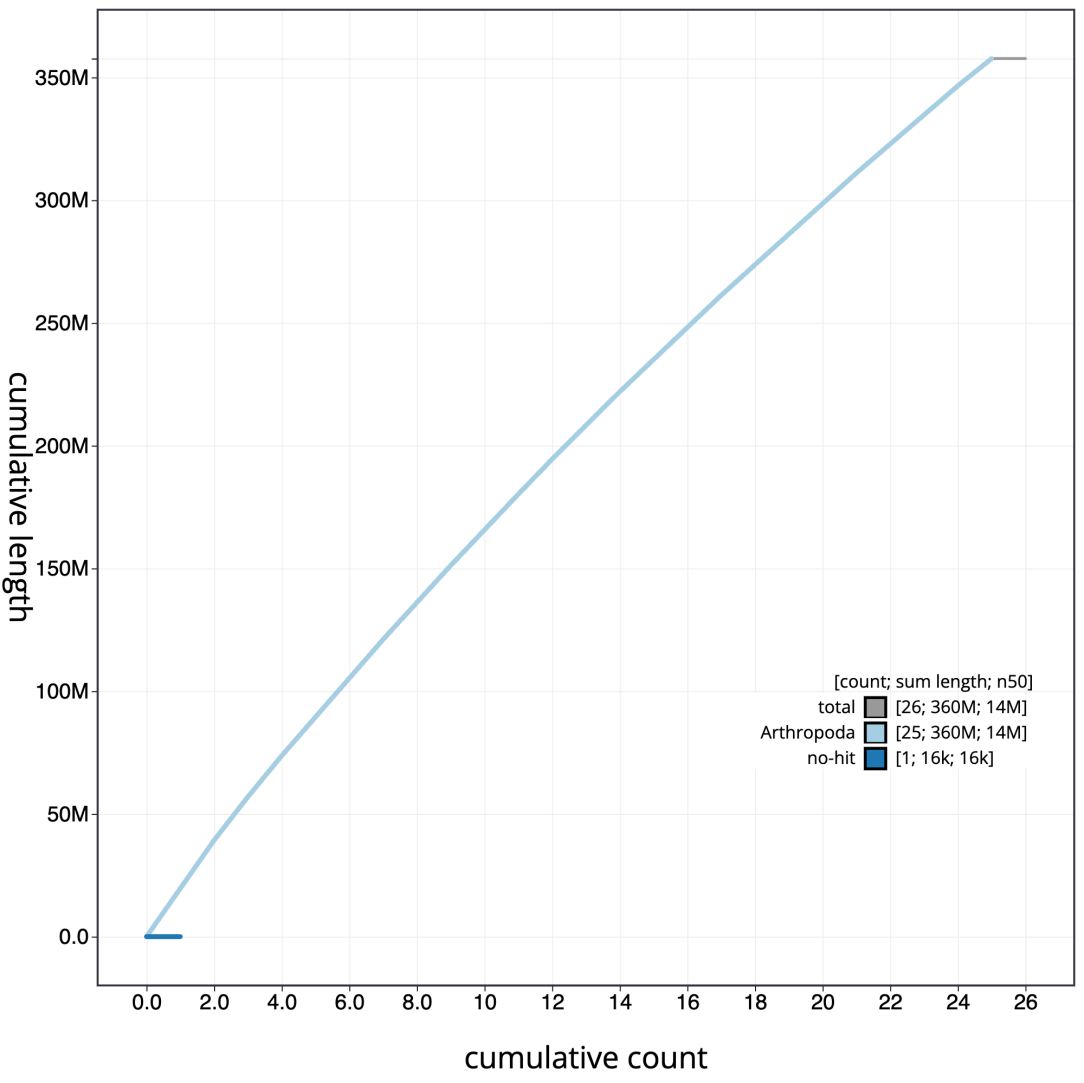
Genome assembly of *Perizoma affinitatum*, ilPerAffn1.1: BlobToolKit cumulative sequence plot. The grey line shows cumulative length for all scaffolds. Coloured lines show cumulative lengths of scaffolds assigned to each phylum using the buscogenes taxrule. An interactive version of this figure is available at https://blobtoolkit.genomehubs.org/view/ilPerAffn1_1/dataset/ilPerAffn1_1/cumulative.

**Figure 5.**
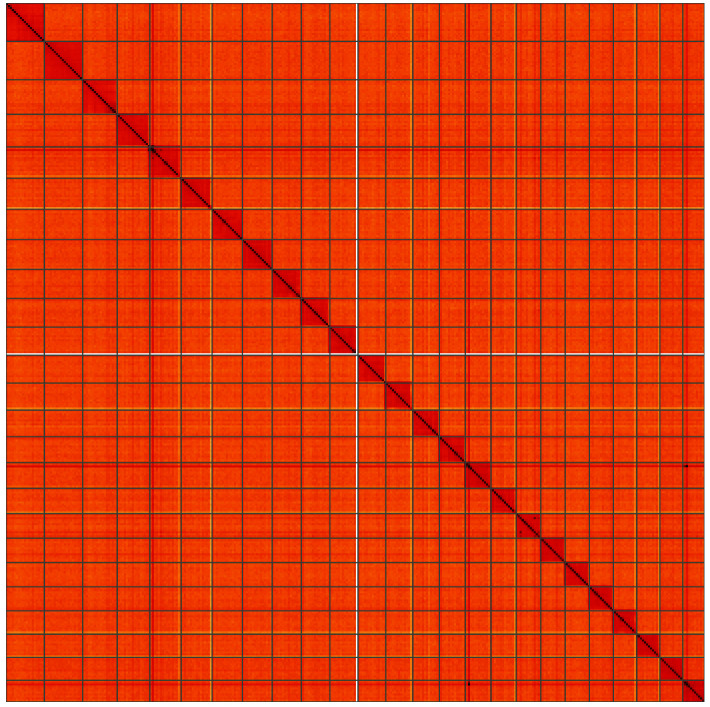
Genome assembly of *Perizoma affinitatum*, ilPerAffn1.1: Hi-C contact map of the ilPerAffn1.1 assembly, visualised using HiGlass. Chromosomes are shown in order of size from left to right and top to bottom. An interactive version of this figure may be viewed at https://genome-note-higlass.tol.sanger.ac.uk/l/?d=X8eLBPMqTZi2IclZKDazZA.

The final assembly has a total length of 357.7 Mb in 25 sequence scaffolds with a scaffold N50 of 14.4 Mb ( Table 1). The snailplot in Figure 2 provides a summary of the assembly statistics, while the distribution of assembly scaffolds on GC proportion and coverage is shown in Figure 3. The cumulative assembly plot in Figure 4 shows curves for subsets of scaffolds assigned to different phyla. Most (99.99%) of the assembly sequence was assigned to 25 chromosomal-level scaffolds, representing 24 autosomes and the Z sex chromosome. Chromosome-scale scaffolds confirmed by the Hi-C data are named in order of size ( Figure 5; Table 2). Chromosome Z was assigned by synteny to *Perizoma flavofasciatum* (GCA_958496245.1). While not fully phased, the assembly deposited is of one haplotype. Contigs corresponding to the second haplotype have also been deposited. The mitochondrial genome was also assembled and can be found as a contig within the multifasta file of the genome submission.

**Table 1.** Genome data for *Perizoma affinitatum*, ilPerAffn1.1.

Project accession data		
Assembly identifier	ilPerAffn1.1	
Species	*Perizoma affinitatum*	
Specimen	ilPerAffn1	
NCBI taxonomy ID	934816	
BioProject	PRJEB63493	
BioSample ID	SAMEA112221815	
Isolate information	ilPerAffn1, male (DNA and Hi-C sequencing)	
Assembly metrics *		*Benchmark*
Consensus quality (QV)	68.9	*≥ 50*
*k*-mer completeness	100.0%	*≥ 95%*
BUSCO **	C:98.2%[S:97.7%,D:0.5%], F:0.4%,M:1.4%,n:5,286	*C ≥ 95%*
Percentage of assembly mapped to chromosomes	99.99%	*≥ 95%*
Sex chromosomes	ZZ	*localised homologous pairs*
Organelles	Mitochondrial genome: 15.9 kb	*complete single alleles*
Raw data accessions		
PacificBiosciences SEQUEL II	ERR11641055	
Hi-C Illumina	ERR11606331	
Genome assembly		
Assembly accession	GCA_961405105.1	
*Accession of alternate * *haplotype*	GCA_961410165.1	
Span (Mb)	357.7	
Number of contigs	53	
Contig N50 length (Mb)	11.9	
Number of scaffolds	25	
Scaffold N50 length (Mb)	14.4	
Longest scaffold (Mb)	19.65	

* Assembly metric benchmarks are adapted from column VGP-2020 of “Table 1: Proposed standards and metrics for defining genome assembly quality” from ( Rhie *et al.*, 2021). ** BUSCO scores based on the lepidoptera_odb10 BUSCO set using version 5.3.2. C = complete [S = single copy, D = duplicated], F = fragmented, M = missing, n = number of orthologues in comparison. A full set of BUSCO scores is available at https://blobtoolkit.genomehubs.org/view/ilPerAffn1_1/dataset/ilPerAffn1_1/busco.

**Table 2.** Chromosomal pseudomolecules in the genome assembly of *Perizoma affinitatum*, ilPerAffn1.

INSDC accession	Chromosome	Length (Mb)	GC%
OY560184.1	1	19.65	36.5
OY560186.1	2	17.71	37.0
OY560187.1	3	16.72	37.0
OY560188.1	4	16.04	37.5
OY560189.1	5	15.79	37.0
OY560190.1	6	15.55	36.5
OY560191.1	7	15.22	37.0
OY560192.1	8	14.93	37.5
OY560193.1	9	14.66	37.0
OY560194.1	10	14.39	36.5
OY560195.1	11	14.27	36.5
OY560196.1	12	13.83	37.0
OY560197.1	13	13.63	36.5
OY560198.1	14	13.24	37.0
OY560199.1	15	13.14	37.0
OY560200.1	16	12.9	37.0
OY560201.1	17	12.61	37.5
OY560202.1	18	12.49	36.5
OY560203.1	19	12.42	36.5
OY560204.1	20	12.27	37.0
OY560205.1	21	11.94	37.0
OY560206.1	22	11.9	37.0
OY560207.1	23	11.68	37.0
OY560208.1	24	11.12	37.5
OY560185.1	Z	19.61	37.0
OY560209.1	MT	0.02	20.0

The estimated Quality Value (QV) of the final assembly is 68.9 with *k*-mer completeness of 100.0%, and the assembly has a BUSCO v5.3.2 completeness of 98.2% (single = 97.7%, duplicated = 0.5%), using the lepidoptera_odb10 reference set ( *n* = 5,286).

Metadata for specimens, barcode results, spectra estimates, sequencing runs, contaminants and pre-curation assembly statistics are given at https://links.tol.sanger.ac.uk/species/934816.

## Methods

### Sample acquisition and nucleic acid extraction

A male *Perizoma affinitatum* (specimen ID NHMUK014536984, ToLID ilPerAffn1) was collected from Gilbert White’s House, Selborne, Hampshire, UK (latitude 51.09, longitude –0.94) on 2021-06-10 using a light trap. The specimen was collected by Gavin Broad, Laura Sivess, Chris Fletcher, Inez Januszczak and Stephanie Holt (Natural History Museum) and identified by Gavin Broad. The specimen was preserved by dry-freezing at –80°C.

Protocols developed by the Wellcome Sanger Institute (WSI) Tree of Life core laboratory have been deposited on protocols.io ( Denton *et al.*, 2023b). The workflow for high molecular weight (HMW) DNA extraction at the WSI includes a sequence of core procedures: sample preparation; sample homogenisation, DNA extraction, fragmentation, and clean-up. In sample preparation, the ilPerAffn1 sample was weighed and dissected on dry ice, setting aside tissue for Hi-C sequencing ( Jay *et al.*, 2023). Tissue from the whole organism was homogenised using a PowerMasher II tissue disruptor ( Denton *et al.*, 2023a).

HMW DNA was extracted in the WSI Scientific Operations core using the Automated MagAttract v2 protocol ( Oatley *et al.*, 2023). HMW DNA was sheared into an average fragment size of 12–20 kb in a Megaruptor 3 system with speed setting 31 ( Bates *et al.*, 2023). Sheared DNA was purified by solid-phase reversible immobilisation ( Strickland *et al.*, 2023): in brief, the method employs a 1.8X ratio of AMPure PB beads to sample to eliminate shorter fragments and concentrate the DNA. The concentration of the sheared and purified DNA was assessed using a Nanodrop spectrophotometer and Qubit Fluorometer and Qubit dsDNA High Sensitivity Assay kit. Fragment size distribution was evaluated by running the sample on the FemtoPulse system.

Protocols developed by the WSI Tree of Life laboratory are publicly available on protocols.io ( Denton *et al.*, 2023b).

### Sequencing

Pacific Biosciences HiFi circular consensus DNA sequencing libraries were constructed according to the manufacturers’ instructions. DNA sequencing was performed by the Scientific Operations core at the WSI on a Pacific Biosciences SEQUEL II instrument. Hi-C data were also generated from whole organism tissue of ilPerAffn1 using the Arima2 kit and sequenced on the Illumina NovaSeq 6000 instrument.

### Genome assembly, curation and evaluation

Assembly was carried out with Hifiasm ( Cheng *et al.*, 2021) and haplotypic duplication was identified and removed with purge_dups ( Guan *et al.*, 2020). The assembly was then scaffolded with Hi-C data ( Rao *et al.*, 2014) using YaHS ( Zhou *et al.*, 2023). The assembly was checked for contamination and corrected as described previously ( Howe *et al.*, 2021). Manual curation was performed using HiGlass ( Kerpedjiev *et al.*, 2018) and PretextView ( Harry, 2022). The mitochondrial genome was assembled using MitoHiFi ( Uliano-Silva *et al.*, 2023), which runs MitoFinder ( Allio *et al.*, 2020) or MITOS ( Bernt *et al.*, 2013) and uses these annotations to select the final mitochondrial contig and to ensure the general quality of the sequence.

A Hi-C map for the final assembly was produced using bwa-mem2 ( Vasimuddin *et al.*, 2019) in the Cooler file format ( Abdennur & Mirny, 2020). To assess the assembly metrics, the *k*-mer completeness and QV consensus quality values were calculated in Merqury ( Rhie *et al.*, 2020). This work was done using Nextflow ( Di Tommaso *et al.*, 2017) DSL2 pipelines “sanger-tol/readmapping” ( Surana *et al.*, 2023a) and “sanger-tol/genomenote” ( Surana *et al.*, 2023b). The genome was analysed within the BlobToolKit environment ( Challis *et al.*, 2020) and BUSCO scores ( Manni *et al.*, 2021; Simão *et al.*, 2015) were calculated.

Table 3 contains a list of relevant software tool versions and sources.

**Table 3.** Software tools: versions and sources.

Software tool	Version	Source
BlobToolKit	4.2.1	https://github.com/blobtoolkit/ blobtoolkit
BUSCO	5.3.2	https://gitlab.com/ezlab/busco
Hifiasm	0.16.1-r375	https://github.com/chhylp123/ hifiasm
HiGlass	1.11.6	https://github.com/higlass/ higlass
Merqury	MerquryFK	https://github.com/ thegenemyers/MERQURY.FK
MitoHiFi	3	https://github.com/ marcelauliano/MitoHiFi
PretextView	0.2	https://github.com/wtsi-hpag/ PretextView
purge_dups	1.2.5	https://github.com/dfguan/ purge_dups
sanger-tol/ genomenote	v1.0	https://github.com/sanger-tol/ genomenote
sanger-tol/ readmapping	1.1.0	https://github.com/sanger-tol/ readmapping/tree/1.1.0
YaHS	1.2a.2	https://github.com/c-zhou/yahs

### Wellcome Sanger Institute – Legal and Governance

The materials that have contributed to this genome note have been supplied by a Darwin Tree of Life Partner. The submission of materials by a Darwin Tree of Life Partner is subject to the **‘Darwin Tree of Life Project Sampling Code of Practice’**, which can be found in full on the Darwin Tree of Life website here. By agreeing with and signing up to the Sampling Code of Practice, the Darwin Tree of Life Partner agrees they will meet the legal and ethical requirements and standards set out within this document in respect of all samples acquired for, and supplied to, the Darwin Tree of Life Project.

Further, the Wellcome Sanger Institute employs a process whereby due diligence is carried out proportionate to the nature of the materials themselves, and the circumstances under which they have been/are to be collected and provided for use. The purpose of this is to address and mitigate any potential legal and/or ethical implications of receipt and use of the materials as part of the research project, and to ensure that in doing so we align with best practice wherever possible. The overarching areas of consideration are:

•      Ethical review of provenance and sourcing of the material

•      Legality of collection, transfer and use (national and international)

Each transfer of samples is further undertaken according to a Research Collaboration Agreement or Material Transfer Agreement entered into by the Darwin Tree of Life Partner, Genome Research Limited (operating as the Wellcome Sanger Institute), and in some circumstances other Darwin Tree of Life collaborators.

## Data Availability

European Nucleotide Archive: *Perizoma affinitatum*. Accession number PRJEB63493; https://identifiers.org/ena.embl/PRJEB63493 ( Wellcome Sanger Institute, 2023). The genome sequence is released openly for reuse. The *Perizoma affinitatum* genome sequencing initiative is part of the Darwin Tree of Life (DToL) project. All raw sequence data and the assembly have been deposited in INSDC databases. The genome will be annotated using available RNA-Seq data and presented through the Ensembl pipeline at the European Bioinformatics Institute. Raw data and assembly accession identifiers are reported in Table 1.

## Author information

Members of the Natural History Museum Genome Acquisition Lab are listed here: https://doi.org/10.5281/zenodo.7139035.

Members of the Darwin Tree of Life Barcoding collective are listed here: https://doi.org/10.5281/zenodo.4893703.

Members of the Wellcome Sanger Institute Tree of Life Management, Samples and Laboratory team are listed here: https://doi.org/10.5281/zenodo.10066175.

Members of Wellcome Sanger Institute Scientific Operations: Sequencing Operations are listed here: https://doi.org/10.5281/zenodo.10043364.

Members of the Wellcome Sanger Institute Tree of Life Core Informatics team are listed here: https://doi.org/10.5281/zenodo.10066637.

Members of the Tree of Life Core Informatics collective are listed here: https://doi.org/10.5281/zenodo.5013541.

Members of the Darwin Tree of Life Consortium are listed here: https://doi.org/10.5281/zenodo.4783558.
