# Supplementary material for: The genome sequence of the Rivulet moth, Perizoma affinitatum (Stephens, 1831)
Source: Wellcome Open Res. 2024 Mar 4;9:127. [Version 1] doi: 10.12688/wellcomeopenres.21016.1 (PMC11427865; doi:10.12688/wellcomeopenres.21016.1)
Supplement: Peer review history

# Reviewer report: Reviewer response for version 1

**DOI:** 10.21956/wellcomeopenres.23251.r94812

**Published:** 2024-09-26

**Authors:** Bhagya c. Thimmappa

**Affiliations:** 1 USDA Agricultural Research Service (Ringgold ID: 17123), Chatsworth, NEW JERSEY, USA

In the article "The genome sequence of the Rivulet moth, *Perizoma affinitatum*," the authors report the nuclear and mitochondrial genomes of male *Perizoma affinitatum*. PacBio HiFi long reads and Hi-C data resulted in a complete nuclear genome assembly of 357.7 Mb distributed in 53 contigs. A BUSCO value of 98.2% using the Lepidoptera data set indicates a nearly complete assembly.

**Minor comments:**

1. There is very little information on mitochondrial genome assembly. It would be nice to have more details in the report section, along with mitochondrial genome annotation.
2. How do the genome and mitochondrial assembly statistics compare to other closely related moths?

Are sufficient details of methods and materials provided to allow replication by others?

Yes

Is the rationale for creating the dataset(s) clearly described?

Yes

Are the datasets clearly presented in a useable and accessible format?

Yes

Are the protocols appropriate and is the work technically sound?

Yes

Reviewer Expertise:

Genomics, Bioinformatics

I confirm that I have read this submission and believe that I have an appropriate level of expertise to confirm that it is of an acceptable scientific standard.

# Reviewer report: Reviewer response for version 1

**DOI:** 10.21956/wellcomeopenres.23251.r94806

**Published:** 2024-09-06

**Authors:** Fahad Alqahtani

**Affiliations:** 1 King Abdulaziz City for Science and Technology, Riyadh, Saudi Arabia

The authors of "The Genome Sequence of the Rivulet Moth, *Perizoma affinitatum* (Stephens, 1831)," have successfully produced a highly complete genome assembly at the chromosomal-level for a male Rivulet moth ( *Perizoma affinitatum*). The genome assembly was performed using two sequencing technologies: Pacific Biosciences SEQUEL II and Hi-C Illumina. The final assembly has a total length of 357.7 Mb and contains 25 chromosomes. The completeness of the genome assembly was evaluated using BUSCO analysis, showing that 98.2% of the common genes are completely present in the lepidoptera_odb10 dataset.

 However, there are a few minor issues that should be addressed:

1. The resolution of Figure 1 should be increased, and the insect should be flipped upside-down
  .
2. The number of contigs is reported as 53, which differs slightly from the number listed in NCBI, which is 52. This difference should be resolved to ensure consistency.

Are sufficient details of methods and materials provided to allow replication by others?

Yes

Is the rationale for creating the dataset(s) clearly described?

Yes

Are the datasets clearly presented in a useable and accessible format?

Yes

Are the protocols appropriate and is the work technically sound?

Yes

Reviewer Expertise:

Bioinformatics

I confirm that I have read this submission and believe that I have an appropriate level of expertise to confirm that it is of an acceptable scientific standard, however I have significant reservations, as outlined above.

# Reviewer report: Reviewer response for version 1

**DOI:** 10.21956/wellcomeopenres.23251.r92806

**Published:** 2024-08-29

**Authors:** Maria Nilsson

**Affiliations:** 1 Senckenberg Biodiversity and Climate Research Centre, Frankfurt am Main, Germany

The nuclear and mitochondrial genome of the Rivulet moth *Perizoma affinitata* has been sequenced.

 The rivulet moth is a common species in southern England and Southern Scotland. It is closely associated with the plant Silene, as the larvae feeds on its seeds. The rivulet has seen a decrease in numbers for the past 35 years.

 The genome was sequenced from two (or one, see below) individuals captured using a light trap. Both pacbio longread sequences as well as HiC was used as a base for the assembly.

 The nuclear assembly of the Rivulet is 357.7Mb large. The assembly is scaffolded into 25 pseudochromosomes which also include the sex chromosome. The scaffold N50 is 14Mb. The QV value is 68.9. Busco score is at 98.2% complete genes. The statistics indicate a very complete assembly, that will be of value for understanding moth evolution as well as to understand the relationship between *Perizoma affinitata* and *Perizoma hydrata.*

**Minor comments:**

 1) At the end of the Background it is stated that the genome is sequenced from two individuals/specimens. However, in the Methods section, there is only mention that one individual was captured and there is nothing that two individuals were used in DNA isolation and sequencing.

 2) For those not knowledgeable in moth biology, I recommend to explain what 'on the wing' as well as what 'bivoltine' means. Both expressions occur in the Background section.

 3) It is stated in the Background that there is a high similarity of *Perizoma affinitata *and *Perizoma hydrata* based on DNA barcodes. For clarity, please add that these are mitochondrial barcodes, as there can also be nuclear barcodes (18S/28S).

Are sufficient details of methods and materials provided to allow replication by others?

Yes

Is the rationale for creating the dataset(s) clearly described?

Yes

Are the datasets clearly presented in a useable and accessible format?

Yes

Are the protocols appropriate and is the work technically sound?

Yes

Reviewer Expertise:

phylogenomics, transposable elements, mitogenomics

I confirm that I have read this submission and believe that I have an appropriate level of expertise to confirm that it is of an acceptable scientific standard, however I have significant reservations, as outlined above.
